# Evaluation of adaptation to visually induced motion sickness based on the maximum cross-correlation between pulse transmission time and heart rate

**DOI:** 10.1186/1743-0003-4-35

**Published:** 2007-09-28

**Authors:** Norihiro Sugita, Makoto Yoshizawa, Makoto Abe, Akira Tanaka, Takashi Watanabe, Shigeru Chiba, Tomoyuki Yambe, Shin-ichi Nitta

**Affiliations:** 1 Graduate School of Engineering, Tohoku University, Aoba 6-6-05, Aramaki, Aoba-ku, Sendai, 980-8579, Japan; 2 Information Synergy Center, Tohoku University, Aoba 6-6-05, Aramaki, Aoba-ku, Sendai, 980-8579, Japan; 3 Faculty of Symbiotic Systems Science, Fukushima University, Kanayagawa 1, Fukushima, 960-1296, Japan; 4 Sharp Corporation, 1-9-2 Nakase, Mihama-ku, Chiba, 261-8520, Japan; 5 Institute of Development, Aging and Cancer, Tohoku University, 4-1 Seiryo-machi, Aoba-ku, Sendai, 980-8575, Japan

## Abstract

**Background:**

Computer graphics and virtual reality techniques are useful to develop automatic and effective rehabilitation systems. However, a kind of virtual environment including unstable visual images presented to wide field screen or a head mounted display tends to induce motion sickness. The motion sickness induced in using a rehabilitation system not only inhibits effective training but also may harm patients' health. There are few studies that have objectively evaluated the effects of the repetitive exposures to these stimuli on humans. The purpose of this study is to investigate the adaptation to visually induced motion sickness by physiological data.

**Methods:**

An experiment was carried out in which the same video image was presented to human subjects three times. We evaluated changes of the intensity of motion sickness they suffered from by a subjective score and the physiological index *ρ*_max_, which is defined as the maximum cross-correlation coefficient between heart rate and pulse wave transmission time and is considered to reflect the autonomic nervous activity.

**Results:**

The results showed adaptation to visually-induced motion sickness by the repetitive presentation of the same image both in the subjective and the objective indices. However, there were some subjects whose intensity of sickness increased. Thus, it was possible to know the part in the video image which related to motion sickness by analyzing changes in *ρ*_max _with time.

**Conclusion:**

The physiological index, *ρ*_max_, will be a good index for assessing the adaptation process to visually induced motion sickness and may be useful in checking the safety of rehabilitation systems with new image technologies.

## Background

In recent years, medical services including rehabilitation programs are changing substantially as results of rapid aging of the population and medical cost inflation. In Japan in 2006, a new law regarding the national health was passed and it fixes a 6 months limit to the coverage for rehabilitation programs. For this reason, more effective and efficient rehabilitation methods are needed to finish a rehabilitation program in a short period of time. In addition, a shortage of manpower for rehabilitation programs grows into a serious problem and therefore it is necessary to automate rehabilitation systems.

In such situations, computer graphics and virtual reality (VR) techniques are useful to develop automatic and effective rehabilitation systems. A system using these techniques is not only safe to use but also attractive for patients, thus some new methods for physical and mental rehabilitation have been proposed [1-5].

However, there are concerns about possible adverse effects of watching novel visual images and experience of VR, such as photosensitive seizures [6,7], visually induced motion sickness (VIMS) [8-11] and eye strain. In particular, when a patient watches an image changed based on real-time information of his head-position, which is sometimes used in VR system, there is a possibility that he watches unexpected images, such as upside-down or rotating, and then he feels VIMS. Since almost all users of the rehabilitation system are aged and/or physically weak, mental or physical stress on them caused by VIMS is considered to be greater than on healthy users.

To prevent these problems, we should establish methods to evaluate the effects of visual stimulation on humans and check a new rehabilitation system prior to use. For this purpose, it would be effective to estimate the autonomic nervous activity by analyzing physiological data such as heart rate and blood pressure [12-14]. A previous study [14] showed that the physiological index, *ρ*_max_, defined as the maximum cross-correlation coefficient between heart rate and blood pressure and whose frequency components are limited to the Mayer wave-related band, is capable of assessing VIMS.

On the other hand, an important feature of motion sickness is the adaptation process. Adaptation occurs with repetitive exposure to the motion that causes the motion sickness [8,9]. The repetitive exposure usually improves motion sickness. This means that the response of *ρ*_max _to the repetitive exposure to the same visual stimulation will be reduced. Most of aged people are inexperienced in watching artificial visual images used in new types of rehabilitation systems and they may feel VIMS at the first time. However, if the symptoms of VIMS improve quickly as the day goes on, patients will be able to use the system.

The purpose of this study is to investigate the adaptation to VIMS by using both subjective and objective indices. We carried out an experiment in which the same video image was presented to subjects three times and analyzed the changes in a subjective score for VIMS and their autonomic nervous activity which was evaluated by continuous estimation of *ρ*_max_.

## Methods

### Experiments

**Figure 1:**
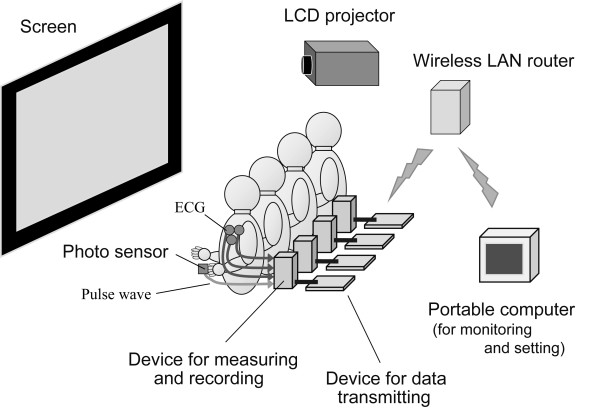
Schematic illustration of the simultaneous experiment with multiple subjects watching the same video image.

Figure 1 shows a schematic illustration of the experiment. A total of 21 healthy subjects participated in the study. Due to the number of devices available for measuring ECG and plethysmogram, a maximum of 11 subjects could watch the same video image simultaneously in any given trial of the experiment. Therefore, the experiment was conducted in two groups. The first had ten subjects (9 males and 1 females; 21.0 ± 2.0 years), and the second 11 (10 males and 1 females; 20.3 ± 1.8 years). They watched the same video image, projected by a LCD projector (resolution: 1024 × 768, brightness: 3250 ANSI lumens), once a day, for three consecutive days. Each subject watched at the same position from the screen and at the same time of each day. The experimental protocol was approved by the University's Internal Review Board and informed consent was obtained from all subjects before the experiment.

The video film presented to the subjects was clipped from a movie made in the U.S.A. in 1999. The movie is notorious for inducing VIMS because it was taken by a handheld camera swayed intentionally to enhance the sensation of reality. To prevent emotional effects, violent scenes included in the film were excluded. The protocol of a given trial was as follows: 1) subjects watched a still picture of a landscape for 5 min as a control; 2) they watched the 15 min video image described above; 3) the same still picture shown in step 1 was watched again for 5 min. After the trial, each subject filled out the Simulator Sickness Questionnaire (SSQ) [15]. The total score (*TS*) on the SSQ is assumed to represent the subjective intensity of VIMS; the higher the *TS*, the more strongly a subject felt motion sickness. In each trial, ECG and finger photo-plethysmogram signals were measured and recorded with a hand-made Mayer-wave analyzer [16]. The resolution of A/D converter and the sampling rate are 12 bit and 1 kHz, respectively.

### Analysis

Heart rate (*HR*) was calculated from the reciprocal of the inter-R-wave interval of the ECG signal. Arterial pulse wave transmission time (*PTT*) was defined as the time interval from the peak of the ECG, R-wave, to the point at which the plethysmogram signal begins to rise. The value of *PTT *is related to blood pressure, as it is dependent on vascular compliance, which is affected by blood pressure [17]. Moreover, instantaneous measurements of *PTT *are much easier to obtain than measurements of blood pressure. Thus, *PTT *was used instead of blood pressure to calculate *ρ*_max _(described in detail below) to evaluate changes in autonomic nervous activity [18].

*HR *and *PTT *were interpolated by cubic spline functions to be continuous-time functions, and were re-sampled every *Δt *= 0.5 s. Each data point was then filtered through a band-pass filter with a bandwidth between 0.08 Hz and 0.1 Hz to extract the Mayer wave component.

To compute the cross-correlation coefficient between these two values, let us *k *denote the discrete time based on *kΔt*. For a simple expression, *x*(*k*) = *PTT*(*k*) and *y*(*k*) = *HR*(*k*). At each second, the cross-correlation function, *ϕ*_*xy*_(*τ*), at lag time *τ *from *x*(*k*) to *y*(*k*) was calculated time-discretely on the basis of 2 min data segments weighted with the Hamming window from -1 min to 1 min. The cross-correlation coefficient, *ρ*_*xy*_(*τ*), was obtained by normalizing *ϕ*_*xy*_(*τ*) with root mean square values of *x*(*k*) and *y*(*k*) as follows:

ρxy(τ)=ϕxy(τ)ϕxx(0)⋅ϕyy(0)
 MathType@MTEF@5@5@+=feaafiart1ev1aaatCvAUfKttLearuWrP9MDH5MBPbIqV92AaeXatLxBI9gBaebbnrfifHhDYfgasaacH8akY=wiFfYdH8Gipec8Eeeu0xXdbba9frFj0=OqFfea0dXdd9vqai=hGuQ8kuc9pgc9s8qqaq=dirpe0xb9q8qiLsFr0=vr0=vr0dc8meaabaqaciaacaGaaeqabaqabeGadaaakeaaiiGacqWFbpGCdaWgaaWcbaGaemiEaGNaemyEaKhabeaakiabcIcaOiab=r8a0jabcMcaPiabg2da9maalaaabaGae8x1dO2aaSbaaSqaaiabdIha4jabdMha5bqabaGccqGGOaakcqWFepaDcqGGPaqkaeaadaGcaaqaaiab=v9aQnaaBaaaleaacqWG4baEcqWG4baEaeqaaOGaeiikaGIaeGimaaJaeiykaKIaeyyXICTae8x1dO2aaSbaaSqaaiabdMha5jabdMha5bqabaGccqGGOaakcqaIWaamcqGGPaqkaSqabaaaaaaa@500F@

where *ϕ*_*xx*_(*τ*) and *ϕ*_*yy*_(*τ*) are auto-correlation functions of *x*(*k*) and *y*(*k*), respectively. Furthermore, the maximum cross-correlation coefficient is defined as physiological index:

*ρ*_max _= max *ρ*_*xy*_(*τ*)

In practice, we obtained *ρ*_max _in a range of *τ *from 0 s to 7 s.

## Results

Two subjects out of 21 complained of VIMS so strongly that they could not complete the trials. Another 5 subjects' data contained artifact and could not be used. Therefore, out of 21 subjects, data from only 14 were available for analysis.

**Figure 2:**
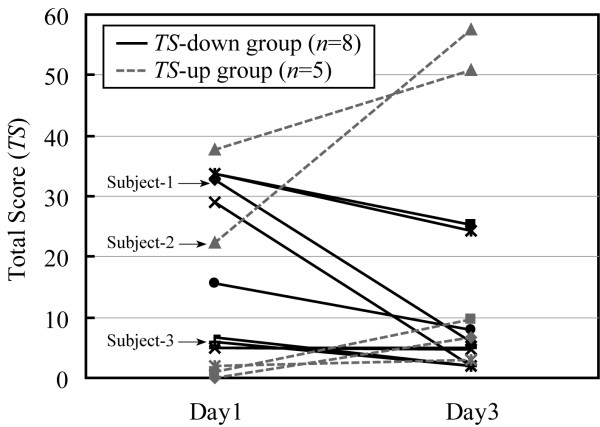
Change, from Day 1 to Day 3, in SSQ total score (*TS*) of 14 subjects.

Figure 2 shows individual changes in these subjects' *TS *on the SSQ from the first day (Day 1) to the third day (Day 3). Based on increase or decrease in the change of *TS *from Day 1 to Day 3, subjects could be divided into two groups: a group of decreased *TS *(*TS*-down; *n *= 8) and a group of increased *TS *(*TS*-up; *n *= 5). A subject belonged to neither group because his *TS *did not change at all between two days. There was no significant difference in *TS *between two groups on Day1, but on Day3, the difference between them was significant (*t*-test, *p *< 0.01).

**Figure 3:**
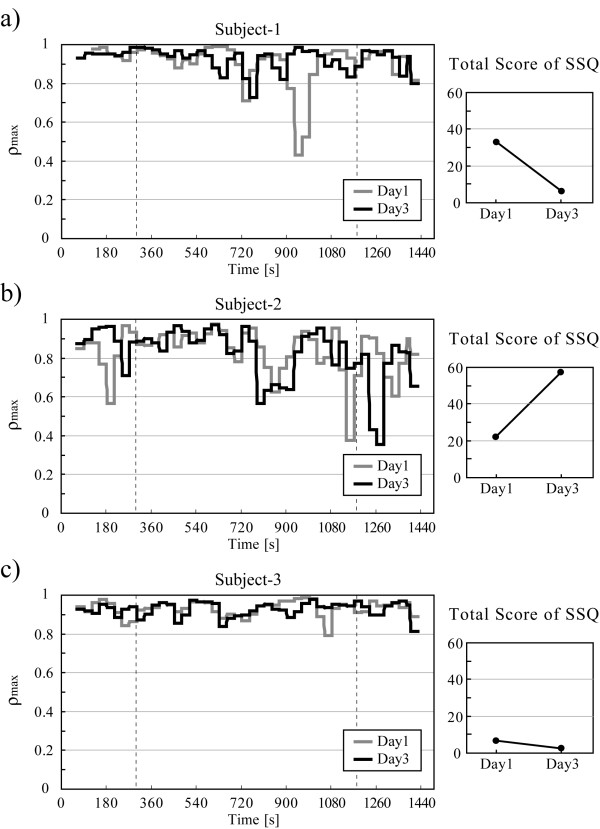
Changes in *ρ*_max _with time (left column) and *TS *(right column) of three subjects on the first and the third days. a) A subject whose *TS *decreased (*TS*-down group), b) one whose *TS *increased (*TS*-up group) and c) one whose *TS *changed little.

Figure 3 shows the change in *ρ*_max _with time of three typical subjects. Subject-1, shown in Fig. 3a, belonged to the *TS*-down group. He reported feeling strong VIMS sensations on the first day, but the intensity of his sickness decreased at the third day. His *ρ*_max _decreased at the same time (around 720 s) on both days but decreased at around 900 s only on the first day. On the other hand, *ρ*_max _of Subject-2 (Fig. 3b), who belonged to the *TS*-up group, decreased considerably in the latter part of the video (720–900 s) and decreased again after watching on both days. Subject-3 (Fig. 3c) did not report feeling much VIMS on any day and there were no apparent changes in his *ρ*_max _on both days.

**Figure 4:**
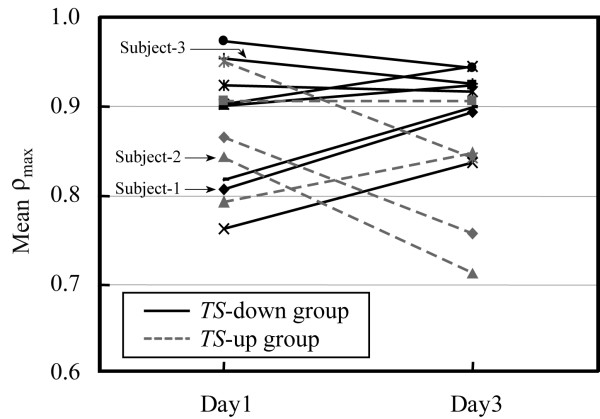
Change, from Day1 to Day3, in *ρ*_max _of individual subjects watching the video at Part-3 (from 750 to 975 s).

To investigate the relationship between the exposure time of visual stimulation and the biological effects of it, we divided the duration of watching the video in quarters, Part-1 (300–525 s), Part-2 (525–750 s), Part-3 (750–975 s) and Part-4 (975–1200 s), and we averaged the *ρ*_max _over each interval with respect to each subject. Figure 4 shows changes in *ρ*_max _of individual subjects from Day 1 to Day 3. Each value represents *ρ*_max _at Part-3. As shown in this figure, the *ρ*_max _increased for 5 of 8 subjects in *TS*-down group and for 2 of 5 subjects in *TS*-up group.

**Figure 5:**
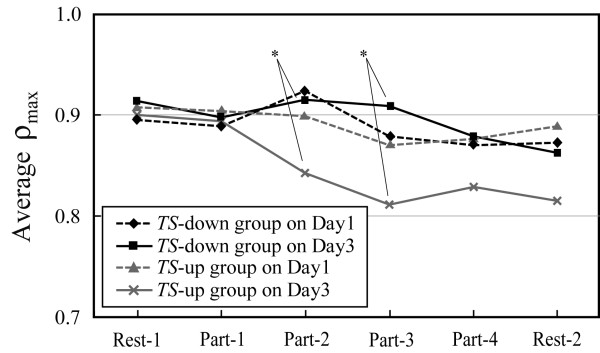
the course of the mean *ρ*_max _changes of *TS*-down and *TS*-up group on Day1 and Day3. *p < 0.05.

Figure 5 shows the course of the mean *ρ*_max _changes of *TS*-down and *TS*-up groups on Day1 and Day3. On Day1, the *ρ*_max _changes of two groups were similar to each other. However, on Day3, the *ρ*_max _of *TS*-up group markedly decreased while watching the video and there was significant difference between the two groups both at Part-2 and Part-3 (*t*-test, *p *< 0.05). In addition, between Day1 and Day3, there was a delay in the time when the *ρ*_max _of *TS*-down group decreased, i.e. the mean value of *ρ*_max _of *TS*-down group decreased at Part-3 on Day1 while decreased at Part-4 on Day3.

## Discussion

As shown in Fig. 2, the subjective score for VIMS (i.e. *TS*) decreased for 8 of the 14 subjects through repetition of watching the same video image. Especially for top 7 subjects with high *TS *on the first day, *TS *of 5 subjects decreased. This change is considered to be adaptation, namely habituation, to the video image and similar results were reported in previous studies [8,9].

The *ρ*_max _of Subject-2 whose *TS *were high both on Day1 and Day3 decreased at the same point (720–900 s; Fig. 3b) on both days while that of Subject-3 whose *TS *were low did not change much (Fig. 3c). These results suggest that the autonomic nervous activity of the subject who actually suffered from VIMS was disturbed by watching the swaying video image; this action affected his baroreflex system, which resulted in a decreased *ρ*_max_. The decrease in *ρ*_max _of Subject-2 at this part of the video agrees with results of previous studies, that it takes about 5 to 10 minutes for the subject to feel the symptoms of VIMS [10,11].

Moreover, the *ρ*_max _of Subject-1 decreased considerably at around 900 s on Day 1 but not on Day 3. This result may correspond to the decrease in his *TS *(i.e. the decrease in the intensity of VIMS) on Day 3. It is suggested that this phenomenon represents an adaptation to VIMS derived from the repetitive exposure to the same swaying video image.

On the other hand, *TS *of the 5 subjects on Day 3 were higher than on Day 1 such as Subject-2 who belonged to *TS*-up group. The changes in *ρ*_max _of these subjects almost correspond to the changes in their *TS*, i.e. *ρ*_max _decreased considerably for 3 of the 5 subjects. Thus it is suggested that VIMS worsened by repetitive exposure to the swaying video image for some subjects. As shown in Fig. 5, the mean value of *ρ*_max _of *TS*-up group decreased from the earlier part of the video on Day 3 than on Day 1 and there was significant difference in the *ρ*_max _between *TS*-down and *TS*-up groups at the middle parts of the video, Part-2 and Part-3. This result indicates that the repetition of watching made some subjects more sensitive to the swaying video and their autonomic nervous system or related physiological mechanism changed easily.

In the result shown in Fig. 4, there were 3 subjects whose *TS *decreased but their *ρ*_max _increased over the three days. However, in these subjects, *ρ*_max _was higher than 0.9 (the maximum value of *ρ*_max _is 1.0) both on Day1 and Day3. Therefore, this result is considered to be caused not by non-adaptation to the swaying video but by no room for *ρ*_max _increasing.

Furthermore, on the first day of the experiment, the subjects might feel nervous or anxiety about the experiment itself, which they had never experienced [19]. For this reason, we must also consider possibility that the low *ρ*_max _was caused by these psychological effects. To test this hypothesis, we should carry out experiments in which the subjects watch just the landscape for the entire 25 min on three days, or we should adjust the subjects to the apparatus before experiments.

In terms of rehabilitation, VIMS induced in using a rehabilitation system not only inhibits effective training but also may harm patients' health. However, by using the evaluation indices such as *TS *and *ρ*_max_, we can check whether a rehabilitation system is safe or not and explore the cause of VIMS. In addition, these indices may be useful in the evaluation of the efficacy of vestibular rehabilitation. Some studies have proposed rehabilitation for the treatment of vestibular disorders with the use of the VR technique [20,21]. Patients with vestibular disorders have symptoms of vertigo, vomiting and disequilibrium. If physiological indices such as *ρ*_max _reflect the intensity of these symptoms, it is possible to evaluate the recovery process of vestibular disorders during a rehabilitation program.

## Conclusion

In this study, a subjective index *TS*, the self-rating score of motion sickness, and a physiological index *ρ*_max_, reflecting autonomic nervous activity, were employed to assess adaptation to VIMS.

In the experiment, the same VIMS-inducing video image was shown to all subjects once each on three consecutive days. The analyses of *TS *and *ρ*_max _for 14 subjects revealed: 1) *TS *decreased from the first day to the third day for more than half of all the subjects (*TS*-down group). 2) There were some subjects whose intensity of VIMS increased as the number of the exposures increased (*TS*-up group). 3) *ρ*_max _of the subjects feeling VIMS decreased at the same point of time on both days. 4) After repetitions of watching the same video image, the *ρ*_max _of *TS*-down group increased at the middle part of the video while that of *TS*-up group decreased.

In the future, we should check whether the adaptation occurs with more repetitions of the visual stimuli because there is a possibility that the number of repetitions in this experiment was too small for adaptation, and should investigate adaptation in the case of watching other video images and VR experience. Moreover, individual differences need to be investigated in more depth. It was reported that not only differences in gender and age [22,23] but also physical and sporting activities [24] affect the susceptibility to motion sickness. These factors should be associated with the differences in *ρ*_max _shown in the present study between the *TS*-down and *TS*-up groups.

## Competing interests

The author(s) declare that they have no competing interests.

## Authors' contributions

All authors read and approved the final manuscript. NS carried out the experiment and drafted the manuscript. MY participated in the design of the study and helped to draft the manuscript. MA carried out the experiment and performed the analysis of data. AT participated in the design of the study. TW, SC, TY and SN helped to draft the manuscript.
